# Rice breeding against sheath blight is now feasible: a breakthrough discovery of* SBRR1*-mediated sheath blight resistance from natural rice germplasm

**DOI:** 10.1007/s44154-025-00266-1

**Published:** 2025-10-13

**Authors:** Qingqing Hou, Xuewei Chen

**Affiliations:** New Cornerstone Science Laboratory, State Key Laboratory of Crop Gene Exploration and Utilization in Southwest China, Sichuan Agricultural University, Chengdu, 611130 Sichuan China

**Keywords:** Rice, *Rhizoctonia solani*, Sheath blight resistance breeding, SBRR1

## Abstract

Sheath blight (ShB), caused by the necrotrophic fungus *Rhizoctonia solani*, is a globally destructive rice disease responsible for significant yield losses. However, the absence of characterized genes conferring high potential resistance to sheath blight within natural rice germplasm constrains resistance breeding. A recent study published in Nature Genetics uncovered the *ShB resistance receptor-like kinase 1* (*SBRR1*) as a key gene associated with disease resistance. *SBRR1-R,* an elite resistance allele mainly presented in *indica* rice and distinguished by a 256-bp promoter insertion, confers strong resistance without obvious yield penalty. *SBRR1* is the first gene with major effects underlying natural variation in sheath blight resistance, offering significant potential for rice breeding. Furthermore, the discovery of the “bHLH57—SBRR1-R—SIP1—Chit3/4” defense module provides fundamental insights into rice immunity and a molecular module with substantial breeding potential.

**Supplementary Information:**

The online version contains supplementary material available at 10.1007/s44154-025-00266-1.

## Main text

Rice sheath blight (ShB) caused by necrotrophic fungus *Rhizoctonia solani* poses one of the most severe threats to global rice production, with 10–50% yield losses annually (Wang et al. 2025). Modern agricultural practices, including deployment of semi-dwarf varieties, high planting density, and excessive nitrogen fertilization, have exacerbated ShB outbreaks by creating favorable conditions for pathogen proliferation (Singh et al. 2019). While current control predominantly relies on chemical fungicides with significant environmental costs, genetic resistance breeding remains the most sustainable solution (Molla et al. 2020; Wang et al. 2025). However, progress has been hindered by the polygenic inheritance of ShB resistance, absence of completely resistant germplasm and technical challenges in resistance phenotyping (Molla et al. 2020; Wang et al. 2023). Although over 60 quantitative trait loci (QTLs) have been identified, none have been successfully isolated through traditional map-based cloning (Molla et al. 2020; Wang et al. 2023). Recent advances in genomics, particularly genome-wide association study (GWAS), have successfully identified several genes associated with ShB resistance, including *OsRSR1* and *OsRLCK5* from natural rice varieties (Wang et al. 2021), as well as the *ROD1* (*SNP1*^*A*^) natural allele which confers enhanced ShB resistance without growth penalty (Gao et al. 2021), but their practical breeding potential remains largely unexplored. In addition, the few genes conferring ShB resistance characterized through reverse genetics approaches often confer partial resistance at the cost of agronomic performance, limiting their breeding utility directly (Nizamani et al. 2025). Consequently, the absence of available ShB resistance genes in the current breeding program presents a major bottleneck for developing resistant rice cultivars (Li et al. 2020).

Compared to biotrophic/hemi-biotrophic pathogens, necrotrophic pathogens present a unique challenge to the host defense system, because they generally employ diverse virulence strategies including cell wall-degrading enzymes and phytotoxins, to directly kill host cells, which has led to poor understanding of host resistance to these pathogens relative to biotrophic/hemi-biotrophic pathogens (Mengiste et al. 2025; Xie et al. 2025). Although reverse genetics approaches have partially uncovered some resistance mechanisms associated with rice resistance to *R. solani*, critical knowledge gaps remain in understanding natural variations in ShB resistance, particularly in how rice perceives this necrotrophic pathogen and mounts effective defense (Cao et al. 2022; Nizamani et al. 2025).

Recently, a remarkable work by Feng et al. (2025) has successfully broken this critical bottleneck by discovering a novel ShB-resistant allele with high breeding potential derived from a natural germplasm. In this study, they identified *SBRR1* (*ShB resistance receptor-like kinase 1*), which encodes a G-type lectin receptor-like protein kinase, as a key gene conferring ShB resistance via GWAS. Within the natural germplasm collection, *SBRR1* exhibits two distinct haplotypes: the favorable haplotype *SBRR1-R* and the unfavorable haplotype *SBRR1-S*. *SBRR1-R* exhibits significantly stronger disease resistance and pathogen responsiveness than *SBRR1-S*, attributable to a 256-bp insertion in its promoter region. Furthermore, *SBRR1-R* has originated from common wild rice *Oryza rufipogon* I, the progenitor of *indica* rice, and shows preferential distribution in *indica* rice varieties from regions with high ShB pressure, suggesting its applicational potential in pathogen defense. Since most *japonica* rice varieties lack the *SBRR1-R* allele, to assess the breeding potential of *SBRR1-R*, they introduced *SBRR1-R* into two high-yield but ShB-susceptible *japonica* cultivars, Taigeng 394 (TG394) and Xudao 3 (XD3), via marker-assisted selection. Near isogenic lines TG394-*SBRR1*^*R*^ and XD3-*SBRR1*^*R*^ exhibit significantly enhanced ShB resistance while maintaining all major agronomic and yield traits. Notably, field trials found that *SBRR1-R* in TG394-*SBRR1*^*R*^ Line reduces yield loss by up to 9.54% compared to TG394 under severe disease pressure, demonstrating the significant breeding value of *SBRR1-R*.

**Fig.1:**
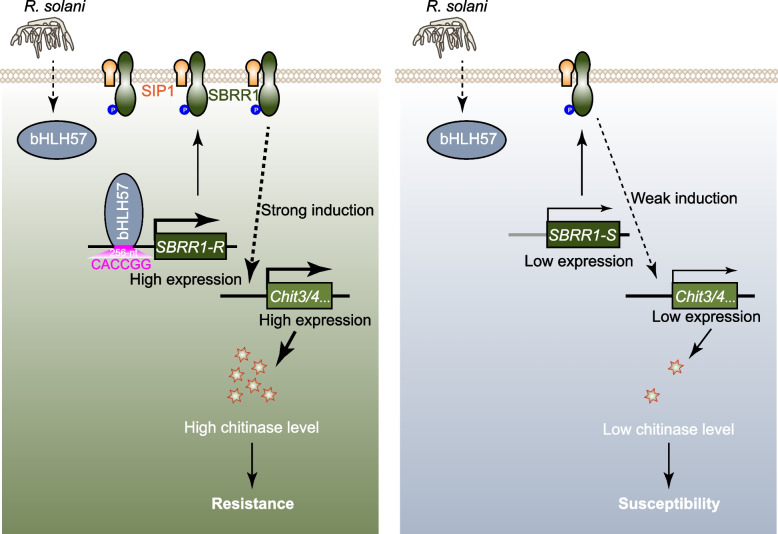
A model for *SBRR1*-mediated ShB resistance**.** In *SBRR1-R* varieties, bHLH57 specifically binds to the ‘CACCGG’ motif of the *SBRR1-R* promoter, elevating *SBRR1* expression. In the cytoplasm, SIP1 interacts with SBRR1 to facilitate effective accumulation of SBRR1 on the plasma membrane. SBRR1 phosphorylation is increased upon *R. solani* attack and is required for ShB resistance. Finally, rapidly elevated expression of chitinase genes accounts for the majority of SBRR1-mediated downstream defense against *R. solani*. In *SBRR1-S* varieties, due to lack of the ‘CACCGG’ motif in the promoter, bHLH57 cannot effectively activate *SBRR1* expression, resulting in lower *SBRR1* expression that leads to less accumulation of chitinases and, thus, disease susceptibility

Feng et al. then comprehensively decoded the multilayered defense network orchestrated by *SBRR1-R* (Fig. 1). Their investigation firstly uncovered that the 256-bp insertion in *SBRR1-R* promoter contains a functional CACCGG cis-element specifically recognized by transcription factor bHLH57, a feature absent in the unfavorable *SBRR1-S* allele, as validated via comprehensive in vitro (yeast one-hybrid and electrophoretic mobility shift assays) and in vivo (chromatin-immunoprecipitation-quantitative PCR) analyses. Subsequent dual-luciferase reporter assays and transgenic tests demonstrated that this interaction forms a pathogen-responsive molecular switch enabling bHLH57 to rapidly activate *SBRR1-R*, conclusively establishing bHLH57 as the master positive regulator of *SBRR1-R*.

At the protein level, this study demonstrated that SBRR1 kinase activity is indispensable for disease resistance, as evidenced by the complete loss of function in kinase-dead KiD transgenic lines. Phosphoproteomic analysis using liquid chromatography-tandem mass spectrometry (LC–MS/MS) revealed that *R. solani* infection strongly induces phosphorylation at TT682/683 residues within the activation loop of SBRR1 kinase domain, establishing phospho-regulation as a critical mechanism for SBRR1-mediated defense signaling. Yeast two-hybrid screening further identified SIP1 (SBRR1-interaction protein 1), an ankyrin-repeat domain-containing protein that facilitates SBRR1 in effective translocation to the plasma membrane. Disruption of this interaction, as seen in SIP1 knockout lines, leads to endoplasmic reticulum retention of SBRR1 and compromises disease resistance, confirming the critical role of proper membrane localization for immune receptor functionality.

Finally, the study definitively established that SBRR1-mediated downstream defense signals against *R. solani* operate through transcriptional activation of downstream chitinase genes *Chit3* and *Chit4*, rigorously validated through transcriptomic analysis, enzymatic activity assays, and transgenic tests. Extensive studies have confirmed that chitinases exert antifungal activity by specifically hydrolyzing β-1,4-glycosidic bonds in chitin polymers to disrupt fungal cell wall integrity and inhibit pathogen growth (Nizamani et al. 2025).

In summary, this study identified *SBRR1-R*, a naturally existing elite allele conferring ShB resistance in rice germplasm with significant breeding potential, and established a molecular ShB defense module containing "bHLH57—SBRR1-R—SIP1—Chit3/4" (Fig. 1). The discovery represents a breakthrough in ShB resistance research, overcoming the persistent challenge of the critical shortage of available genes confer resistance to ShB with breeding potential. Notably, while SBRR1 exhibits kinase activity critical for ShB resistance, its phosphorylation mechanisms and direct substrates remain to be determined. Furthermore, although SIP1-dependent membrane localization is crucial for SBRR1 function, its ability to regulate nuclear *Chit3/4* expression likely involves intermediate signaling components. Further investigation is needed to elucidate how SBRR1 triggers a membrane-to-nucleus signaling cascade to transmit defense signals.

Although over 20 genes associated with ShB resistance have been identified (Table S1), most were characterized via reverse genetics approaches, and their underlying defense mechanisms remain poorly understood. Moreover, the lack of natural resistance alleles with proven breeding potential has hindered ShB resistance breeding. The identification of *SBRR1-R* fills this gap, providing a foundation for further exploration of ShB resistance mechanisms and breeding strategies. The "bHLH57—SBRR1-R—SIP1—Chit3/4" module not only offers a framework for identifying novel interactors regulating SBRR1 kinase activity but also facilitates the exploration of SBRR1 homologs in related crops. Importantly, the demonstrated breeding value of SBRR1-R in japonica rice highlights its utility as an exemplary case of natural allele mining for resistance breeding.

Receptor-like kinases (RLKs) are central components of plant immunity, functioning in the perception of external signals and activation of downstream defense pathways (Fan et al. 2018). Several RLKs have been identified in resistance against necrotrophic fungal pathogens. The *SBRR1-R*-mediated defense module uncovers a previously unrecognized layer of regulation, where an RLK integrates transcriptional control (bHLH57) with chitinase activation (Chit3/4) to restrict pathogen invasion. These findings reveal a membrane-to-nucleus signaling cascade that expands our understanding of RLK-mediated necrotrophic resistance and plant immune responses.

Rice has accumulated abundant natural variations during evolution and domestication. These variations underlie agronomic traits and breeding resources. Notably, *Japonica* rice is more susceptible to ShB than *indica* (Jia et al. 2011). The *SBRR1-R* allele, predominantly found in *indica* varieties, has been successfully introgressed into ShB-susceptible *japonica* cultivars TG394 and XD3 through marker-assisted selection. The resulting near-isogenic Lines exhibit enhanced ShB resistance without yield penalty and suffered markedly reduced losses under severe disease pressure. These results demonstrate the potential of broadening ShB resistance across rice subspecies. For breeding applications, breeders can utilize the co-segregating 256-bp insertion marker for efficient selection, combining *SBRR1-R* with complementary resistance QTLs to enhance durability, and targeting *SBRR1* in japonica to broaden resistance. With its proven efficacy and multiple deployment strategies, we affirm that ShB-resistant rice breeding is now practically feasible. *SBRR1-R* deployment in breeding programs promises to reduce reliance on chemical fungicides, enhance food security, and contribute to sustainable rice production.

## Supplementary Information

Supplementary Material 1. Table S1. Main regulators of ShB resisitance in rice.

## Data Availability

Not applicable.

## Abbreviations

ShB: Sheath blight
SBRR1: ShB resistance receptor-like kinase 1
QTLs: Quantitative trait loci
GWAS: Genome-wide association study
TG394: Taigeng 394
XD3: Xudao 3
bHLH57: Basic helix-loop helix 57
LC–MS/MS: Liquid chromatography-tandem mass spectrometry
SIP1: SBRR1-interacting protein
Chit3: Chitinase 3
Chit4: Chitinase 4
