# Supplementary material for: Rice breeding against sheath blight is now feasible: a breakthrough discovery of SBRR1-mediated sheath blight resistance from natural rice germplasm
Source: Stress Biol. 2025 Oct 13;5(1):59. doi: 10.1007/s44154-025-00266-1 (PMC12515794; doi:10.1007/s44154-025-00266-1)
Supplement: Supplementary file 1 — Supplementary Material 1. Table S1. Main regulators of ShB resisitance in rice. [file 44154_2025_266_MOESM1_ESM.docx]

**Rice breeding against sheath blight is now feasible: A breakthrough discovery of *SBRR1*-mediated sheath blight resistance from natural rice germplasm**

**Supplementary Information**

| **Table S1 Main regulators of ShB resisitance in rice** | | |  |  |  |
| --- | --- | --- | --- | --- | --- |
| **Gene** | **Accession number** | **Encoding protein** | **Regulation Direction** | **Molecular Mechanism** | **Reference** |
| *OsWRKY30* | LOC_Os08g38990 | WRKY transcription factor | Positve | *OsWRKY30* promotes JA accumulation and PR gene expression to increase ShB resistance | Peng et al., 2012 |
| *OsWRKY4* | LOC_Os03g55164 | WRKY transcription factor | Positve | *OsWRKY4* enhances ShB resistance via JA/ET-dependent signaling | Wang et al., 2015 |
| *OsWRKY80* | LOC_Os03g63810 | WRKY transcription factor | Positve | *OsWRKY80* enhances ShB resistance by directly activating OsWRKY4 | Peng et al., 2016 |
| *OsWRKY53* | LOC_Os05g27730 | WRKY transcription factor | Negative | OsWRKY53 and OsGT1 activate OsSWEET2a and OsSWEET3a to suppress ShB resistance | Yang et al., 2023 |
| *OsGT1* | LOC_Os03g10210 | HD-Zip transcription factor | Negative | OsWRKY53 and OsGT1 activate OsSWEET2a and OsSWEET3a to suppress ShB resistance | Yang et al., 2023 |
| *OsDOF11* | LOC_Os02g47810 | Dof Transcription Factor | Positve | *OsDOF11* contributes to ShB resistance via activation of SWEET14, reducing apoplastic sugar availability for R. solani. | Kim et al., 2021 |
| *LPA1/OsIDD14* | LOC_Os03g13400 | IDD transcription factor | Positve | *LPA1* enhances resistance by activating PIN1a-mediated auxin IAA accumulation. | Sun et al., 2019 |
| *OsIDD10* | LOC_Os04g47860 | IDD transcription factor | Negative | *OsIDD10* inhibits rice resistance to ShB by activating the ethylene receptor OsETR2 | Li et al., 2025 |
| *OsERF65* | LOC_Os07g42510 | ERF transcription factor | Negative | *OsERF65* negatively modulates ShB resistance by inhibiting ROS scavenging ability | Xie et al., 2023 |
| *OsHSFA4d* | LOC_Os05g45410 | HSF transcription factor | Negative | OsHSFA4a promotes Os*CsLF6* expression to suppress ShB resistance | Fang et al., 2025 |
| *OsbHLH25* | LOC_Os01g09990 | bHLH transcription factor | Positve | *OsbHLH25* senses H2O2 to enhance resistance to ShB resistance | Liao et al., 2025 |
| *OsSWEET2a* | LOC_Os01g36070 | SWEET sugar transporter | Negative | OsSWEET2a and OsSWEET3a efflux sugar into the extracellular space to provide nutrients for the pathogens | Yang et al., 2023 |
| *OsSWEET3a* | LOC_Os05g12320 | SWEET sugar transporter | Negative | OsSWEET2a and OsSWEET3a efflux sugar into the extracellular space to provide nutrients for the pathogens | Yang et al., 2023 |
| *OsSWEET11* | LOC_Os08g42350 | SWEET sugar transporter | Negative | OsSWEET11 efflux sugar into the extracellular space to provide nutrients for the pathogens | Gao et al., 2018 |
| *OsSWEET14* | LOC_Os11g31190 | SWEET sugar transporter | Positve | OsSWEET14 reduce sugar content in the apoplasm to inhibit R. solani growth | Kim et al., 2021 |
| *OsACBP5* | LOC_Os03g14000 | acyl-CoA-binding protein | Positve | *OsACBP5* enhance ShB resistance might via JA-dependent pathways | Panthapulakkal Narayanan et al., 2020 |
| *OsDEP1* | LOC_Os09g26999 | Gγ subunit | Negative | *OsDEP1* negatively regulates rice resistance to ShB by modulated OsIDD14 and OsIDD10 interactions to regulate Os*PIN1a* and Os*ETR2* | Zhu et al., 2025 |
| *OsPIN1a* | LOC_Os02g50960 | PIN-FORMED auxin exporter | Positve | *OsPIN1a* positively regulates ShB resistance via auxin signaling | Sun et al., 2019 |
| *OsETR2* | LOC_Os04g08740 | Ethylene Receptor | Negative | *OsETR2*inhibits ethylene signaling to compromise resistance to ShB | Li et al., 2025 |
| *OsAMT1;1* | LOC_Os04g43070 | ammonium transporter | Positve | *OsAMT1;*1 activates nitrogen utilization and NH+4 -dependent ethylene-related genes to enhance ShB resistance | Wu et al., 2022 |
| *OsBGL2* | LOC_Os11g47820 | β-glucanase | Positve | *OsBGL2* Promotes Resistance to ShB by Inhibiting the Permeability of Plasmodesmata | Zhou et al., 2023 |
| *OsPP2A-1* | LOC_Os06g37660 | protein phosphatase | Positve | *OsPP2A-1* enhances rice resistance to ShB via *PBZ1* and *PR1b* induction | Lin et al., 2021 |
| *ROD1* | LOC_Os06g03810 | Ca2+ sensor | Negative | *ROD1* supresses ShB resistance by promotes ROS scavenging | Gao et al., 2021 |
| *UMP1* | LOC_Os03g38720 | proteasome maturation factor | Positve | *OsUMP1* increases H_2_O_2_ accumulation to enhance ShB resistance | Hu et al., 2023 |
| *OsSGR* | LOC_Os09g36200 | chlorophyll-degrading Mg2+-dechelatase | Negative | *OsSGR* confers resistance to ShB through cytokinin content | Xie et al., 2025 |
| *OsCKX7* | LOC_Os02g12780 | cytokinin oxidase/dehydrogenase | Negative | *OsCKX7* confers resistance to ShB through cytokinin content | Xie et al., 2025 |
| *OsRSR1* | LOC_Os11g12340 | NLR | Positve | *OsRSR1* enhances resistance to ShB by regulating ROS homeostasis. | Wang et al., 2021 |
| *OsRLCK5* | LOC_Os01g02390 | RLCK | Positve | *OsRLCK5* enhances resistance to ShB by regulating ROS homeostasis. | Wang et al., 2021 |
| *SBRR1* | LOC_Os11g10290 | G-type LecRLK | Positve | *SBRR1* confers ShB resistance by upregulating chitinase genes | Feng et al., 2025 |

**References**

Fang Y, Liao H, Wei Y, Yin J, Cha J, Liu X, Chen X, Chen L, Ma Z, Zhang J et al (2025) OsCDPK24 and OsCDPK28 phosphorylate heat shock factor OsHSFA4d to orchestrate abiotic and biotic stress responses in rice. Nat Commun 16: 6485. https://doi.org/10.1038/s41467-025-61827-6.

Feng Z, Gao P, Wang G, Kang H, Zhao J, Xie W, Chen R, Ju R, Wang X, Wei Z et al (2025) Natural variation in SBRR1 shows high potential for sheath blight resistance breeding in rice. Nat Genet 57:2004-2016. https://doi.org/10.1038/s41588-025-02281-4.

Gao M, He Y, Yin X, Zhong X, Yan B, Wu Y, Chen J, Li X, Zhai K, Huang Y et al (2021) Ca2+ sensor-mediated ROS scavenging suppresses rice immunity and is exploited by a fungal effector. Cell 184:5391-5404. https://doi.org/10.1016/j.cell.2021.09.009

Gao Y, Zhang C, Han X, Wang Z, Ma L, Yuan D, Wu J, Zhu X, Liu J, Li D et al (2018) Inhibition of OsSWEET11 function in mesophyll cells improves resistance of rice to sheath blight disease. Mol Plant Pathol 19:2149-2161. https://doi.org/10.1111/mpp.12689.

Hu X, Shen S, Wu J, Liu J, Wang H, He J, Yao Z, Bai Y, Zhang X, Zhu Y et al (2023) A natural allele of proteasome maturation factor improves rice resistance to multiple pathogens. Nat Plants 9:228-237. https://doi.org/10.1038/s41477-022-01327-3

Kim P, Xue C, Song H, Gao Y, Feng L, Li Y, and Xuan Y (2021) Tissue-specific activation of DOF11 promotes rice resistance to sheath blight disease and increases grain weight via activation of SWEET14. Plant Biotechnol J 19:409-411. https://doi.org/10.1111/pbi.13489.

Li Z, Chen H, Yuan D, Jiang X, Li Z, Wang S, Zhou T, Zhu H, Bian Q, Zhu X et al (2025) IDD10-NAC079 transcription factor complex regulates sheath blight resistance by inhibiting ethylene signaling in rice. J Adv Res 71:93-106. https://doi.org/10.1016/j.jare.2024.05.032.

Liao H, Fang Y, Yin J, He M, Wei Y, Zhang J, Yong S, Cha J, Song L, Zhu X et al (2025) Rice transcription factor bHLH25 confers resistance to multiple diseases by sensing H2O2. Cell Res 35:205-219. https://doi.org/10.1038/s41422-024-01058-4.

Lin Q, Chu J, Kumar V, Yuan D, Li Z, Mei Q, Xuan Y (2021) Protein Phosphatase 2A Catalytic Subunit PP2A-1 Enhances Rice Resistance to Sheath Blight Disease. Front Genome Ed 3: 632136. https://doi.org/10.3389/fgeed.2021.632136.

Panthapulakkal Narayanan S, Lung S.C, Liao P, Lo C, Chye M.L (2020) The overexpression of OsACBP5 protects transgenic rice against necrotrophic, hemibiotrophic and biotrophic pathogens. Sci Rep 10:14918. https://doi.org/10.1038/s41598-020-71851-9.

Peng X, Hu Y, Tang X, Zhou P, Deng X, Wang H, Guo Z (2012) Constitutive expression of rice *WRKY30* gene increases the endogenous jasmonic acid accumulation, PR gene expression and resistance to fungal pathogens in rice. Planta 236:1485-1498. https://doi.org/10.1007/s00425-012-1698-7.

Peng X, Wang H, Jang JC, Xiao T, He H, Jiang D, Tang X (2016) OsWRKY80-OsWRKY4 Module as a Positive Regulatory Circuit in Rice Resistance Against *Rhizoctonia solani*. Rice 9:63. https://doi.org/10.1186/s12284-016-0137-y.

Sun Q, Li T, Li D, Wang Z, Li S, Li D, Han X, Liu J, Xuan Y (2019) Overexpression of *Loose Plant Architecture 1* increases planting density and resistance to sheath blight disease via activation of *PIN-FORMED 1a* in rice. Plant Biotechnol J 17:855-857. https://doi.org/10.1111/pbi.13072.

Wang A, Shu X, Jing X, Jiao C, Chen L, Zhang J, Ma L, Jiang Y, Yamamoto N, Li S et al (2021) Identification of rice (Oryza sativa L.) genes involved in sheath blight resistance via a genome-wide association study. Plant Biotechnol J 19:1553-1566. https://doi.org/10.1111/pbi.13569.

Wang H, Meng J, Peng X, Tang X, Zhou P, Xiang J, Deng X (2015) Rice WRKY4 acts as a transcriptional activator mediating defense responses toward *Rhizoctonia solani*, the causing agent of rice sheath blight. Plant Mol Biol 89:157-171. https://doi.org/10.1007/s11103-015-0360-8.

Wu X, Yuan D, Chen H, Kumar V, Kang S, Jia B, Xuan Y (2022) Ammonium transporter 1 increases rice resistance to sheath blight by promoting nitrogen assimilation and ethylene signalling. Plant Biotechnol J 20:1085-1097. https://doi.org /10.1111/pbi.13789.

Xie W, Cao W, Lu S, Zhao J, Shi X, Yue X, Wang G, Feng Z, Hu K, Chen Z et al (2023) Knockout of transcription factor *OsERF65* enhances ROS scavenging ability and confers resistance to rice sheath blight. Mol Plant Pathol 24:1535-1551. https://doi.org/10.1111/mpp.13391.

Xie W, Xue X, Wang Y, Zhang G, Zhao J, Zhang H, Wang G, Li L, Wang Y, Shan W et al (2025) Natural mutation in Stay-Green (OsSGR) confers enhanced resistance to rice sheath blight through elevating cytokinin content. Plant Biotechnol J 23:807-823. https://doi.org /10.1111/pbi.14540.

Yang S, Fu Y, Zhang Y, Yuan D, Li S, Kumar V, Mei Q, Xuan Y (2023) *Rhizoctonia solani* transcriptional activator interacts with rice WRKY53 and grassy tiller 1 to activate SWEET transporters for nutrition. J Adv Res 50:1-12. https://doi.org/10.1016/j.jare.2022.10.001.

Zhou T, He Y, Han X, Sun Q, Xuan Y (2023) β-Glucanase Family Genes Promote Resistance to Sheath Blight in Rice by Inhibiting the Permeability of Plasmodesmata. J Agric Food Chem 71:9667-9676. https://doi.org/10.1021/acs.jafc.3c01127.

Zhu H, Zhou T, Guan J, Li Z, Yang X, Li Y, Sun J, Xu Q, Xuan Y (2025) Precise genome editing of *Dense and Erect Panicle 1* promotes rice sheath blight resistance and yield production in japonica rice. Plant Biotechnol J 23:1832-1846. https://doi.org/10.1111/pbi.70010.
